# High Sugar Induced RCC2 Lactylation Drives Breast Cancer Tumorigenicity Through Upregulating MAD2L1

**DOI:** 10.1002/advs.202415530

**Published:** 2025-03-27

**Authors:** Bowen Zheng, Yunhao Pan, Fengyuan Qian, Diya Liu, Danrong Ye, Bolin Yu, Seng Zhong, Wenfang Zheng, Xuehui Wang, Baian Zhou, Yuying Wang, Lin Fang

**Affiliations:** ^1^ Department of Breast and Thyroid Surgery Shanghai Tenth People's Hospital School of Medicine Tongji University Shanghai 200070 China; ^2^ Department of Breast Surgery Shanghai Second People's Hospital Shanghai 200011 China; ^3^ Department of Breast Surgery The First Affiliated Hospital of Wenzhou Medical University Wenzhou 325000 China; ^4^ Department of Surgery The University of Hong Kong‐Shenzhen Hospital Shenzhen 518053 China

**Keywords:** cell division, high sugar diet, MAD2L1, RCC2, SERBP1

## Abstract

Lactylation is a novel post‐translational modification mediated by lactate, widely present in the lysine residues of both histone and non‐histone proteins. However, the specific regulatory mechanisms and downstream target proteins remain unclear. Herein, it is demonstrated that the RCC2 protein may serve as a critical link between material metabolism and cell division, promoting the rapid proliferation of breast cancer under high glucose conditions. Mechanistically, the activation of glycolysis leads to an increase in lactate. Then, acyltransferase KAT2A mediates RCC2 lactylation at K124, which assists RCC2 in recruiting free SERBP1, thereby stabilizing MAD2L1 mRNA. The lactylation of RCC2 mediates the activation of the cellular MAD2L1 signaling pathway and contributes to the progression of breast cancer. A small molecule inhibitor slows down cell proliferation by binding to the RCC2 active pocket and specifically blocking RCC2 lactylation. The findings elucidate the mechanism behind the upregulation of MAD2L1 in murine tumors associated with a high‐sugar diet as reported in prior study and suggest a novel therapeutic strategy of targeting RCC2 lactylation to restrict the rapid proliferation of breast cancer cell in a high‐lactate microenvironment.

## Graphical abstract

This study finds that KAT2A mediates the lactylation modification of the non‐histone protein RCC2 at K124, facilitating the recruitment of free SERBP1 and aiding in the formation of the initial SERBP1‐MAD2L1 complex. SERBP1 enhances the stability of MAD2L1 mRNA and promotes cellular proliferation. Targeting RCC2 lactylation to restrict the rapid proliferation of breast cancer represents a novel proof‐of‐concept strategy.

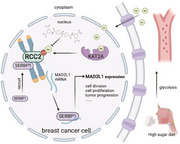

## 1 Introduction

Breast cancer is the most commonly diagnosed malignant tumor among women, and its incidence has been on the rise in recent years.^[^ ^1^ ^]^ Extensive research has been conducted on the diagnosis and treatment of breast cancer,^[^ ^2^ ^]^ however, the molecular mechanisms underlying its occurrence and progression remain shrouded in mystery. Lactic acid, a product of glycolysis, is found in nearly all types of cells. Under the Warburg effect, lactic acid levels are notably abundant in rapidly proliferating tumor cells.^[^ ^3^, ^4^ ^]^ Numerous studies have demonstrated that lactate promotes the proliferation of tumor cells,^[^ ^5^ ^]^ mediates resistance to chemotherapy,^[^ ^6^ ^]^ and contributes to the establishment of a pro‐tumor microenvironment.^[^ ^7^ ^]^ However, the mechanisms by which intracellular lactate is converted into signals that drive tumor growth remain unclear. Recently, lactylation has been identified as a novel post‐translational modification of proteins, found in both histones and non‐histones lysine residues, offering new insights into the non‐metabolic functions of lactate.^[^ ^8^ ^]^

The process of mitotic division in mammalian cells is a highly conserved and intricate biological mechanism that necessitates precise regulation to ensure the accurate allocation of genetic information to daughter cells. MAD2L1 is a pivotal component of the mitotic spindle assembly checkpoint. During the early stages of mitosis, MAD2L1 interacts with CDC20 to inhibit APC activity, resulting in a loss of cohesion and the polymerization function of sister chromatids as the cell transitions from metaphase to anaphase. The normal function of MAD2L1 involves its accumulation at the kinetochore, generating a “wait” signal that prevents the cell from progressing to the later phases of the cell cycle until the spindle microtubules are appropriately aligned with the kinetochores on each chromosome.^[^ ^9^, ^10^ ^]^ However, excessive expression of MAD2L1 can lead to the inactivation of APC/C, resulting in uncontrolled cell division. In various tumors, MAD2L1 signaling is excessively activated and associated with poor prognosis.^[^ ^11^ ^]^ Previous studies have observed a correlation between high‐sugar diets and elevated MAD2L1 expression in pancreatic tumor tissues within animal models,^[^ ^12^ ^]^ however, the precise regulatory mechanisms remain unclear.

RCC2, which is also referred to as TD‐60, was initially recognized as a unique autoantigen specific to human mitosis, chilling out right at the centromeres of prophase chromosomes.^[^ ^13^ ^]^ RCC2 exhibits a characteristic distribution of the chromosomal passenger complex (CPC) within mitotic cells.^[^ ^14^ ^]^ Similar to the RCC1 proteins within the same family, RCC2 has been reported to function as a guanine exchange factor (GEF) that regulates the activity of the G protein RalA, with its deficiency leading to significant mitotic defects in cells.^[^ ^15^ ^]^ Additionally, the study indicates that in gliomas, RCC2 interacts with the transcription factor BACH1 to modulate the expression of HK2.^[^ ^16^ ^]^ The function of RCC2 in carcinoma has garnered significant interest. Dysregulated RCC2 expression correlates with tumor development, unfavorable prognosis, and resistance to chemotherapy.^[^ ^17^, ^18^ ^]^

Here, we reported the RCC2 protein as a substrate for lactylation modifications, linking material metabolism and cell division, which in turn promotes the proliferation of breast cancer. Specifically, under high glucose conditions, the acyltransferase KAT2A mediates the RCC2 site 124 lysine lactylation. The lactylated RCC2 recruits free SERBP1 protein to the vicinity of chromatin, facilitating stabilization of MAD2L1 mRNA, and thereby activating cell division. Our findings elucidate the mechanism of MAD2L1 signaling activation in tumor tissues under high‐sugar dietary conditions. RCC2K124lac may serve as a potential therapeutic target for progressive breast cancer.

## 2 Results

### 2.1 High Sugar Increases Lactate Levels and Upregulates Breast Tumor MAD2L1

**Figure 1:**
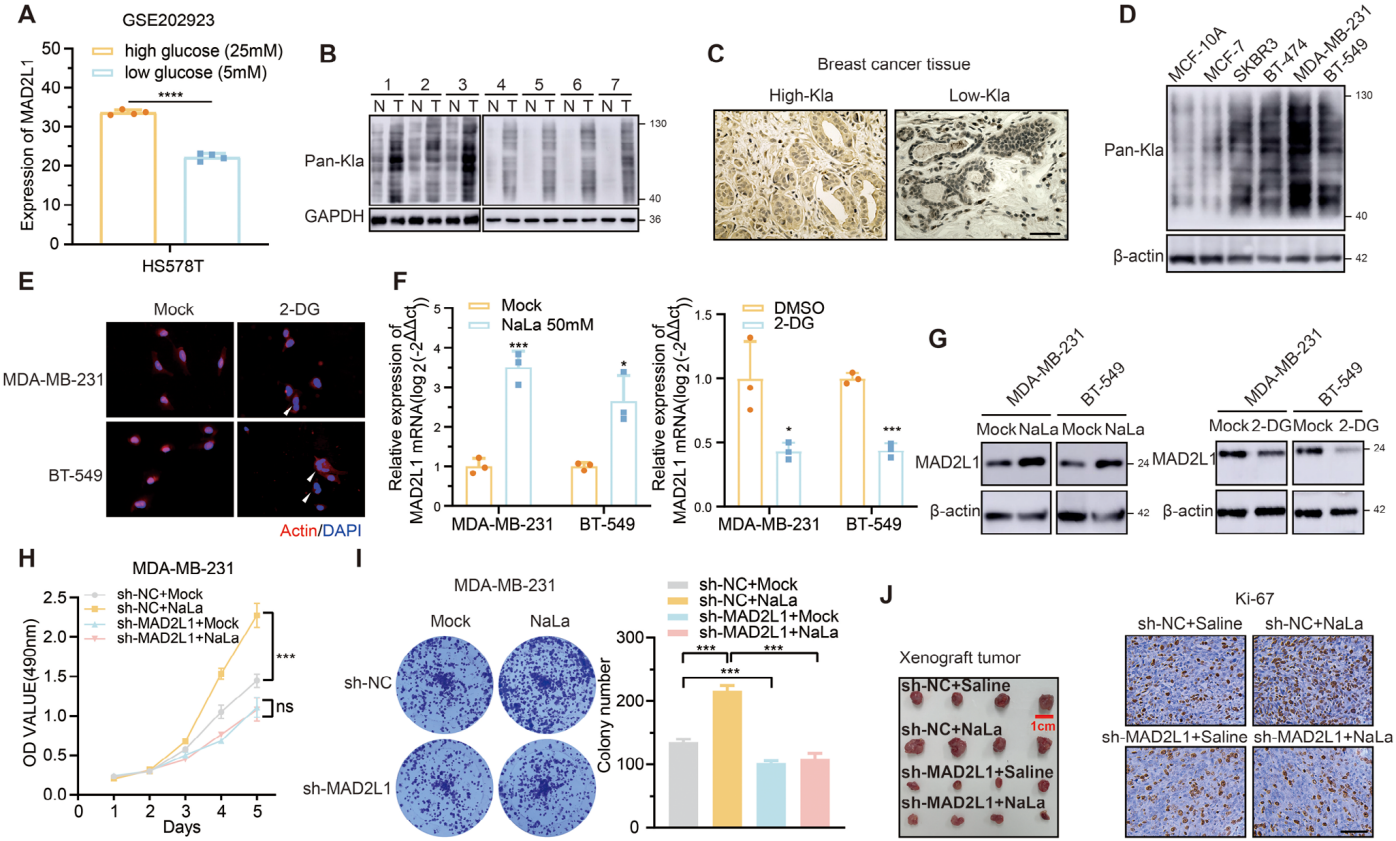
Lactate upregulates MAD2L1 expression in breast cancer. A) MAD2L1 expression level under high‐glucose (25 mm) or low‐glucose (5 mm) culture conditions in HS578T cell in GSE202923. B) Western blot analysis of global lactylation level in seven pairs of normal tissue (N) and breast cancer tissue (T). C) IHC analysis of global lactylation level in 40 breast cancer, and divide them into two groups, high‐Kla and low‐Kla. D) Western blot analysis of global lactylation level in normal mammary epithelial MCF‐10A cell and breast cancer cells with different molecular subtypes. E) IF analysis after 24 h 10 mm 2‐DG treatment. Scale bars, 40 µm. F) PCR assays of MAD2L1 expression level after NaLa and 2‐DG treatment. G) Western blot analysis of MAD2L1 expression level after NaLa and 2‐DG treatment. H,I) MTT and colony formation assays were used to assess the proliferation capacity of the MDA‐MB‐231 cell when MAD2L1 was knocked down with or without 50 mm NaLa treatment. J) MDA‐MB‐231 cells stably expressing sh‐NC and sh‐MAD2L1 were subcutaneously injected into the nude mice. These mice were treated with or without NaLa (120 mg kg^−1^ once a day). Tumor images and Ki‐67 staining were shown. Scale bars, 100 µm. Data are presented as mean ± SEM. *p < 0.05, **p < 0.01, ***p < 0.001, ****p < 0.0001.

Given the existing in vivo evidence indicating that elevated glucose levels are associated with the upregulation of MAD2L1 expression in tumor tissues,^[^ ^12^ ^]^ it is crucial to determine whether this alteration occurs within the tumor cells or in the surrounding microenvironment. Notably, in dataset GSE202923,^[^ ^19^ ^]^ breast cancer cells HS578T cultured in low‐glucose conditions exhibited a significant downregulation of MAD2L1 (**Figure** **1**A), suggesting that glucose metabolism may influence the MAD2L1 signaling pathway in tumor cells. Given that lactic acid is the end product of anaerobic glycolysis and accumulates significantly in tumors, coupled with reports are indicating that a high‐glucose diet can induce hyperlactatemia,^[^ ^20^ ^]^ we hypothesized that the upregulation of MAD2L1 and activation of proliferation‐mediated by high glucose might be associated with the increase in lactic acid. Consistent with previous reports, we observed that low‐glucose culture conditions reduced the abundance of L‐lactate within breast cancer cells (Figure S1A, Supporting Information). Compared to adjacent non‐cancerous tissue, breast cancer tissue exhibited elevated lactate levels and increased global protein lactylation (Figure 1B; Figure S1B, Supporting Information). We performed immunohistochemical staining using Pan‐Kla antibodies on 40 breast cancer tumor tissues, which were stratified into two groups (Figure 1C): high‐Kla and low‐Kla (20 samples per group). Correlation analysis with clinical data revealed a significant association between elevated lactylation levels and increased tumor size (**Table** **1**). Furthermore, Pan‐Kla levels were analyzed in breast cancer cell lines and normal breast epithelial cells, with MDA‐MB‐231 cell showing the highest levels (Figure 1D). To investigate rapid cell proliferation mechanisms under high lactate conditions, we selected MDA‐MB‐231 and BT‐549 cell lines with shorter doubling times for subsequent studies. NaLa treatment significantly promoted breast cancer cell proliferation, whereas 2‐DG‐mediated glycolysis inhibition suppressed proliferative activity (Figure S1C, Supporting Information). Additionally, we noted that cells treated with 2‐DG exhibited a higher proportion of multinucleation (Figure 1E), which may be associated with a disruption in the cell division process. This evidence suggests that lactate and lactylation levels are correlated with tumor proliferation and cell division. NaLa treatment induced marked upregulation of MAD2L1 at the protein and transcriptional levels, whereas 2‐DG markedly reduced MAD2L1 expression (Figure 1F,G). Notably, NaLa supplementation in low‐glucose medium almost completely reversed the suppression of MAD2L1 expression caused by glucose deprivation (Figure S1D, Supporting Information). We also observed the expression of the lactate transporter SLC16A1 and the newly identified lactate transferase AARS are positively correlated with MAD2L1 (Figure S1E, Supporting Information).^[^ ^21^, ^22^ ^]^ These findings strongly suggest that lactate accumulation serves as a key mechanistic driver of high glucose‐induced MAD2L1 upregulation. In rescue experiments using MAD2L1‐silenced tumor cells, NaLa treatment showed minimal capacity to enhance proliferation (Figure 1H,I). The size of the xenograft tumors and the Ki‐67 staining also indicate that silencing MAD2L1 significantly slows down cellular proliferation in vivo, regardless of whether NaLa intervention is applied (Figure 1J). Collectively, these results demonstrate that high‐glucose conditions promote lactate accumulation, which drives proliferative activation in breast cancer via MAD2L1 upregulation.

**Table 1:** The relationship between the IHC score of Pan‐Kla level and clinicopathological variables in BC patients.

Patients’ Characteristics	Total	Pan‐Kla level		
		Low [*n = 20*]	High [*n = 20*]	*p‐*value^a)^
Age				
<60	17	8	9	0.749
≥60	23	12	11	
TNM stage				
I and II	22	13	9	0.204
III and IV	18	7	11	
Tumor size(cm)				
≤2	21	15	6	0.004^b)^
>2	19	5	14	
Lymph node metastasis				
negative	31	16	15	0.705
positive	9	4	5	
Distant metastasis				
No	37	18	19	0.508
Yes	3	2	1	

The relationships between the IHC score of Pan‐Kla level and patients’ clinicopathologic characteristics were analyzed via the Chi‐square test or Fisher's exact test.

^a)^ *p* < 0.05;

^b)^ *p* < 0.01;

^c)^ *p* < 0.001.

### 2.2 RCC2 Acts as a Lactylation Substrate to Upregulate MAD2L1

**Figure 2:**
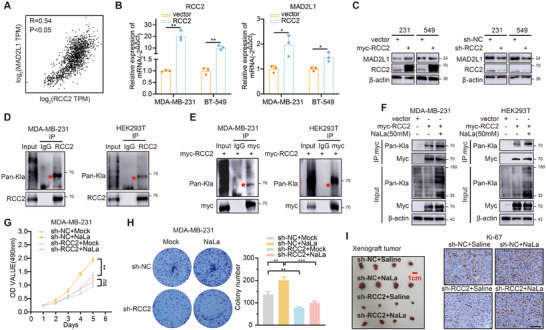
RCC2 serves as a lactylation substrate to upregulate MAD2L1. A) Correlation analysis of the expression of RCC2 and MAD2L1 in breast cancer tissues from the TCGA database. (http://gepia.cancer‐pku.cn/) B) PCR assays of MAD2L1 expression level when RCC2 was overexpressed. C) Western blot analysis of MAD2L1 protein level when RCC2 was overexpressed or knocked down. D) Detection of endogenous lactylation of RCC2 protein in MDA‐MB‐231 and HEK293T cells. E) Detection of lactylation of exogenous RCC2 protein in MDA‐MB‐231 and HEK293T cells after transfection plasmids. F) Lactylation level of exogenous RCC2 with or without 50 mm NaLa treatment. G,H) MTT and colony formation assays were used to assess the proliferation capacity of the MDA‐MB‐231 cell when RCC2 was knocked down with or without 50 mm NaLa treatment. I) MDA‐MB‐231 cells stably expressing sh‐NC and sh‐RCC2 were subcutaneously injected into the nude mice. These mice were treated with or without NaLa (120 mg kg^−1^ once a day). Tumor images and Ki‐67 staining were shown. Scale bars, 100 µm. Data are presented as mean ± SEM. *p < 0.05, **p < 0.01, ***p < 0.001, ****p < 0.0001.

Next, we investigated the molecular mechanism by which lactate metabolism upregulates MAD2L1. The role of lactylation, a recently identified post‐translational modification,^[^ ^8^ ^]^ in tumors remains largely unknown. Initially, we hypothesized that MAD2L1 might be a substrate for lactylation, influencing cellular division and proliferation. We transfected MDA‐MB‐231 cells with overexpression plasmids and performed exogenous immunoprecipitation. However, no specific lactylation band for MAD2L1 (≈25 kDa) was detected in the immunoprecipitated fractions (Figure S2A, Supporting Information), suggesting that MAD2L1 is not a direct substrate for lactylation. In the TCGA breast cancer cohort, RCC2 was identified as a gene co‐expressed with MAD2L1 (Figure **2**A). RCC2 has been previously shown to regulate cell proliferation.^[^ ^15^, ^23^ ^]^ RCC2 is overexpressed in breast cancer and correlates with shorter disease‐free survival (Figure S2B,C, Supporting Information). We found a significant correlation between the expression of RCC2 and MAD2L1 in breast cancer (R = 0.54) (Figure 2A). STRING database analysis supports the association between RCC2 and MAD2L1 (Figure S2D, Supporting Information), but no direct protein interaction has been confirmed by mass spectrometry. Therefore, we investigated whether RCC2 regulates MAD2L1 RNA expression. Overexpression of RCC2 in MDA‐MB‐231 and BT‐549 cells significantly upregulated MAD2L1 expression (Figure 2B), which was confirmed at the protein level (Figure 2C). Conversely, RCC2 knockdown downregulated MAD2L1 (Figure 2C). Then we have formulated a hypothesis, could RCC2 protein serve as a lactylation substrate, linking glucose metabolism and the MAD2L1 pathway? To confirm the existence of lactylation modifications on RCC2, we conducted both endogenous and exogenous immunoprecipitation in MDA‐MB‐231 and HEK293T cells. Specific lactylation bands were detected at the molecular weight of RCC2 in both endogenous and exogenous immunoprecipitates (Figure 2D,E). Treatment with 50 mm NaLa increased RCC2 lactylation (Figure 2F). However, NaLa treatment did not significantly affect the mRNA and protein levels of RCC2 (Figure S2E,F, Supporting Information). Additionally, Low glucose (5 mM) and 2‐DG treatment both downregulated RCC2 lactylation in MDA‐MB‐231 cells (Figure S2G,H, Supporting Information). These results confirm that RCC2 undergoes lactylation. We generated stable RCC2‐knockdown (sh‐RCC2) and control (sh‐NC) MDA‐MB‐231 cells using lentiviral vectors. RCC2 silencing partially reversed NaLa‐induced proliferation in MTT and colony formation assays (Figure 2G,H), which was confirmed by xenograft tumor size and Ki‐67 staining (Figure 2I). 2‐DG treatment also reversed the pro‐proliferative effect of RCC2 overexpression (Figure S2I, Supporting Information). In summary, these results indicate that RCC2, as a lactylation substrate, regulates the MAD2L1 pathway and promotes breast cancer proliferation in a high lactate environment.

### 2.3 KAT2A Mediates RCC2 Lactylation at K124

**Figure 3:**
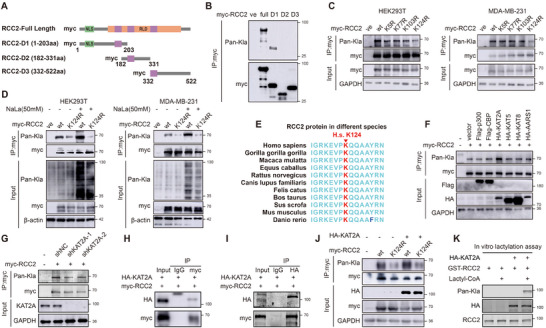
KAT2A mediates RCC2 lactylation at K124. A) Plasmid schematic. B) Lysine lactylation of various peptide segments of the RCC2 protein was detected in HEK293T. C) Western blot analysis of exogenous RCC2 lactylation when transfected specific mutation plasmids. D) Exogenous RCC2 lactylation level when transfected RCC2 K124R plasmids with or without 50 mm NaLa treatment. E) Conservation analysis of RCC2 protein sequences in different species. F) Exogenous RCC2 lactylation level when a series of acyltransferases were overexpressed in MDA‐MB‐231. G) Exogenous RCC2 lactylation level when KAT2A was knocked down in MDA‐MB‐231. H,I) CoIP and WB analysis of RCC2 and KAT2A. J) Exogenous RCC2 lactylation level when KAT2A was overexpressed with or without RCC2 K124R mutation in MDA‐MB‐231. K) In vitro lactylation assay.

Next, we will further investigate the mechanisms of RCC2 lactylation. To identify the precise lactylation sites on RCC2, we constructed plasmids containing myc‐tagged truncated sequences of RCC2 (**Figure** **3**A). Following the transfection of a series of plasmids into HEK293T cells, we enriched the corresponding components using myc‐tag antibodies, and it was found that only the D1 (1‐203aa) subsection was detected by antibodies targeting lactylated lysines (Figure 3B). We analyzed lactylation mass spectrometry data from human tissues reported in the literature and found that peptide segments containing K5, K77, K103, and K124 in the RCC2 protein were identified as lactylated.^[^ ^24^, ^25^, ^26^ ^]^ These lysines are located in the D1 segment, which is consistent with our results (Figure 3B). Subsequently, we constructed a series of point mutation plasmids tagged with myc and conducted IP and western blotting assays. Significant downregulation of RCC2 lactylation was observed only when the RCC2 K124R mutant plasmid was transfected (Figure 3C). Furthermore, in two cell lines transfected with the RCC2 K124R plasmid, despite an overall increase in lactylation levels following NaLa treatment, the lactylation level of RCC2 was markedly suppressed (Figure 3D). Sequence analysis confirmed that K124 is conserved across species (Figure 3E). These results indicate that RCC2 protein undergoes lactylation at K124.

The lactylation modification of substrate proteins depends on upstream acyltransferases.^[^ ^27^ ^]^ To identify acyltransferases regulating RCC2 lactylation, we conducted a series of screenings based on previous studies,^[^ ^28^ ^]^ overexpressing representative KAT family members, including the newly identified lactyltransferase AARS1.^[^ ^5^ ^]^ Only KAT2A overexpression significantly increased RCC2 lactylation (Figure 3F; Figure S2J, Supporting Information), while KAT2A silencing downregulated RCC2 lactylation (Figure 3G; Figure S2K, Supporting Information). KAT2A has been reported to mediate protein acetylation,^[^ ^29^ ^]^ succinylation,^[^ ^30^ ^]^ and crotonylation.^[^ ^31^ ^]^ Recent studies indicate that KAT2A also functions as a lactyltransferase.^[^ ^32^ ^]^ Like RCC2, KAT2A primarily localizes to the nucleus,^[^ ^30^ ^]^ suggesting their potential interaction opportunities. Exogenous Co‐IP confirmed the interaction between KAT2A and RCC2 (Figure 3H,I). Subsequently, rescue experiments demonstrated KAT2A's specificity for RCC2K124lac (Figure 3J). Additionally, we expressed and purified human RCC2 and KAT2A, then co‐incubated them with Lactyl‐CoA. In vitro, lactylation assays confirmed that KAT2A mediates RCC2 lactylation (Figure 3K). Therefore, we conclude that KAT2A is a bona fide lactyltransferase for RCC2.

### 2.4 RCC2 Upregulates MAD2L1 Depend on SERBP1

**Figure 4:**
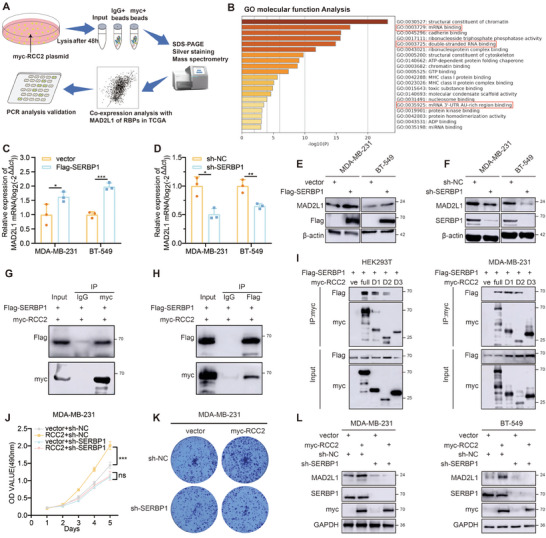
RCC2 interacts with SERBP1 to regulate MAD2L1. A) Schematic diagram of the job screening process. B) GO molecular function enrichment analysis of RCC2's potential interacting proteins. C,D) PCR assays of MAD2L1 expression level when SERBP1 was overexpressed or knocked down. E,F) Western blot analysis of MAD2L1 expression level when SERBP1 was overexpressed or knocked down. G,H) CoIP and WB analysis of RCC2 and SERBP1. I) CoIP and Western blot analysis of exogenous SERBP1 and segmented RCC2. J,K) MTT and colony formation assays were used to assess the proliferation capacity of the SERBP1‐silenced MDA‐MB‐231 cell with or without RCC2 overexpression. L) Western blot analysis of MAD2L1 expression level in SERBP1‐silenced cell with or without RCC2 overexpression. Data are presented as mean ± SEM. *p < 0.05, **p < 0.01, ***p < 0.001, ****p < 0.0001.

We next explored the mechanisms by which RCC2 regulates MAD2L1 expression. Since no studies report RCC2 directly binding DNA or RNA, we hypothesized that RCC2 interacts with other proteins to regulate MAD2L1 expression. To identify downstream proteins of RCC2, we performed the work as shown (**Figure** **4**A). RCC2 was immunoprecipitated from lysates of RCC2‐overexpressing MDA‐MB‐231 cells, followed by silver staining and mass spectrometry (Figure S3A,B and Table S3, Supporting Information). We noted many RNA‐binding proteins were identified among the RCC2‐interaction proteins. GO enrichment analysis revealed that, in addition to chromatin‐related functions, the interacting proteins were enriched in mRNA and ribosomal protein binding (Figure 4B). This suggested that RCC2 might regulate MAD2L1 mRNA post‐transcriptional processing through RBPs. To identify specific RBPs, we analyzed their correlation with MAD2L1 in the TCGA database, identifying highly correlated RBPs (R > 0.45, including NONO, SERBP1, HNRNPR, HNRNPU) (Figure S3C, Supporting Information). Overexpression of these RBPs showed that only SERBP1 increased MAD2L1 mRNA levels (Figure 4C; Figure S3D, Supporting Information). Using shRNA to silence SERBP1 also inhibited the expression of MAD2L1 (Figure 4D), which has been confirmed at the protein level (Figure 4E,F). Both forward and reverse IP experiments, alongside explorations of protein STRING data, support the physical interaction between RCC2 and SERBP1 (Figure 4G,H; Figure S3E, Supporting Information). Co‐transfection of RCC2 fragments and full‐length SERBP1 revealed that RCC2's D1 and D2 domains interact with SERBP1 (Figure 4I), suggesting an N‐terminal interaction. SERBP1 has been reported to play a critical role in cell division.^[^ ^33^ ^]^ We hypothesized that RCC2 regulates the MAD2L1 pathway and cell division through SERBP1. As expected, SERBP1 silencing reversed RCC2‐mediated proliferation activation (Figure 4J,K; Figure S3F,G, Supporting Information) and MAD2L1 upregulation (Figure 4L). RCC2 overexpression did not alter SERBP1 protein levels, indicating that the interaction does not affect SERBP1 degradation (Figure S3H, Supporting Information). Given that RCC2 is primarily localized to chromatin,^[^ ^14^ ^]^ while SERBP1 shuttles between the nucleus and cytoplasm as an RNA transport protein. We hypothesize that RCC2 recruits SERBP1 to chromatin, facilitating the processing of newly transcribed pre‐mRNA. We observed that the overexpression of RCC2 slightly increased the levels of SERBP1 in the nuclear fraction (Figure S3I, Supporting Information), suggesting altered SERBP1 subcellular localization. Here, we conclude that the RCC2 protein interacts with SERBP1 to upregulate MAD2L1.

### 2.5 SERBP1 Binds to and Stabilizes MAD2L1 mRNA

**Figure 5:**
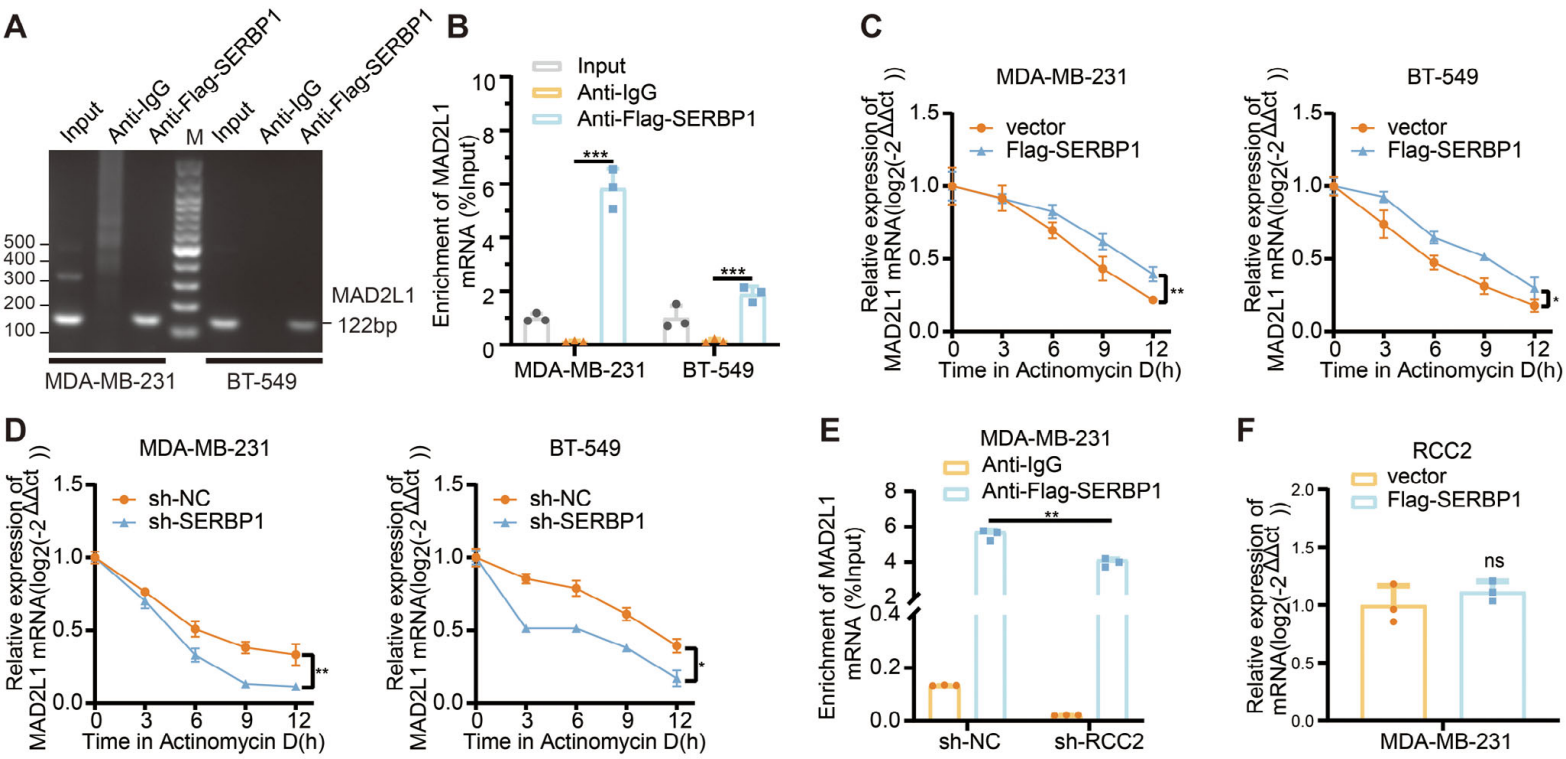
SERBP1 binds to and stabilizes MAD2L1 mRNA. A) RIP assay followed by nucleic acid gel electrophoresis analysis. B) RIP assays followed by PCR analysis. C,D) PCR assay showing the expression of MAD2L1 when SERBP1 was overexpressed or knocked down in MDA‐MB‐231 and BT‐549 cells at 0, 3, 6, 9, and 12 h Act D treatment. E) PCR assay of the level of MAD2L1 mRNA enriched by the same Flag antibodies when RCC2 was knocked down in MDA‐MB‐231. F) PCR assays of RCC2 expression level when SERBP1 was overexpressed in MDA‐MB‐231. Data are presented as mean ± SEM. *p < 0.05, **p < 0.01, ***p < 0.001, ****p < 0.0001.

Given that we have established the regulatory role of SERBP1 in MAD2L1 regulation at both RNA and protein levels, we further investigated the precise mechanism involved. We transfected breast cancer cells with Flag‐SERBP1 plasmids and conducted an exogenous RIP experiment. Subsequent nucleic acid gel electrophoresis and qRT‐PCR indicated an interaction between the SERBP1 protein and MAD2L1 mRNA (**Figure** **5**A,B). Recently published RIP‐seq data from KSHV‐transformed metanephric mesenchymal precursor cells further corroborated this conclusion.^[^ ^34^ ^]^ Given the existing literature indicating that SERBP1 binds to the 3′UTR of mRNA and stabilizes it,^[^ ^34^, ^35^ ^]^ we conducted actinomycin D experiments. Under conditions in which new RNA synthesis was inhibited, the overexpression of SERBP1 extended the half‐life of MAD2L1 mRNA (Figure 5C). Conversely, the knockdown of SERBP1 resulted in a shortened half‐life of MAD2L1 mRNA (Figure 5D). Interestingly, silencing RCC2 appeared to inhibit the ability of SERBP1 to bind MAD2L1 mRNA, as evidenced by a reduced amount of MAD2L1 mRNA enriched by antibodies under identical conditions (Figure 5E). In cells overexpressing SERBP1, no upregulation of RCC2 expression was observed, which rules out the possibility of SERBP1 acting as a potential upstream regulator of RCC2 (Figure 5F). These results suggest that SERBP1 can enhance the stability of MAD2L1 mRNA and that the RCC2 protein may influence the initial assembly of the SERBP1‐MAD2L1 complex.

### 2.6 Lactylation Modifications Facilitate RCC2 to Recruit Free SERBP1

**Figure 6:**
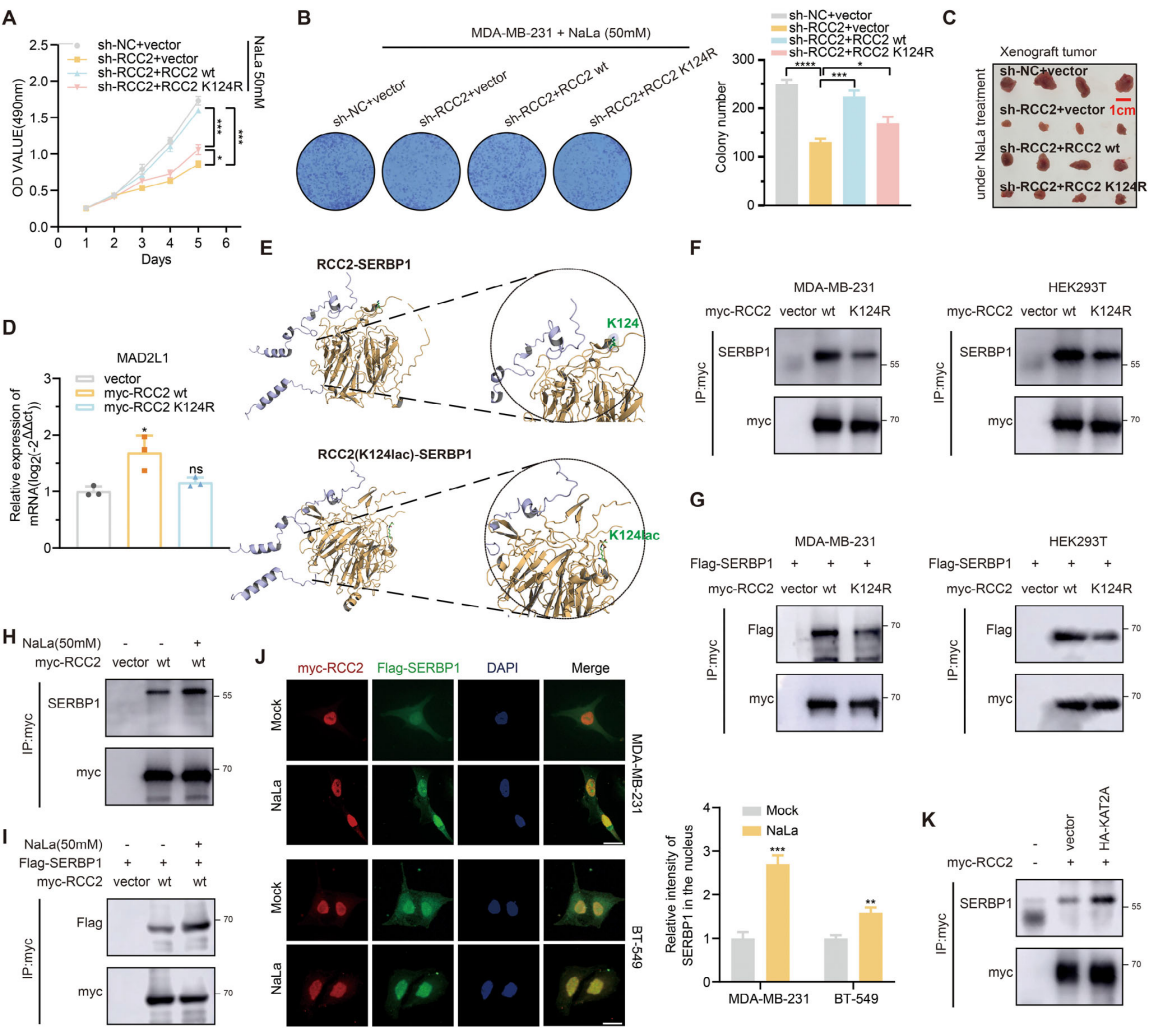
RCC2 lactylation helps recruit free SERBP1. A,B) Cell proliferation of control (sh‐NC) or RCC2‐silenced MDA‐MB‐231 cell overexpressed with control vector or wt‐RCC2 (RCC2 wt.) or mut‐RCC2 (RCC2 K124R) in the presence of NaLa were measured in MTT and colony formation assays. C) Xenograft tumor images of control (sh‐NC) or RCC2‐silenced MDA‐MB‐231 cell overexpressed with control vector or wt‐RCC2 (RCC2 wt.) or mut‐RCC2 (RCC2 K124R) under Intermittent NaLa treatment. D) PCR assays of MAD2L1 expression level when overexpressed with control vector or wt‐RCC2 or mut‐RCC2 in MDA‐MB‐231. E) Molecular docking of RCC2 and SERBP1 using autodock software when incorporation or absence of a lactyl group at RCC2 site 124 lysine. F,G) Endogenous and exogenous IP and WB analysis of the interaction intensity between RCC2 and SERBP1 when overexpressed with wt‐RCC2 or mut‐RCC2. H,I) Endogenous and exogenous IP and WB analysis of the interaction intensity between RCC2 and SERBP1 when with or without 50 mm NaLa treatment. J) Immunofluorescence imaging and statistical analysis of cellular localization of RCC2 and SERBP1 proteins with or without 50 mm NaLa treatment. Scale bars, 20 µm. K) IP and WB analysis of the interaction intensity between RCC2 and SERBP1 when overexpressed with the control vector or KAT2A in MDA‐MB‐231. Data are presented as mean ± SEM. *p < 0.05, **p < 0.01, ***p < 0.001, ****p < 0.0001.

We next examined whether RCC2 K124 lactylation (RCC2K124lac) plays a role in breast cancer cell proliferation. First, we silenced RCC2 using shRNA to eliminate endogenous RCC2's influence on proliferation. Consistent with previous reports, RCC2 silencing significantly reduced cell proliferation.^[^ ^16^ ^]^ In NaLa‐treated cells, overexpression of wild‐type RCC2, but not the K124R mutant, restored proliferation inhibited by RCC2 deficiency (**Figure** **6**A,B). To assess RCC2K124lac's oncogenic effects in vivo, we intraperitoneally injected NaLa to mimic a high‐lactate tumor microenvironment. Consistent with in vitro results, xenograft tumor proliferation inhibited by RCC2 silencing was restored only by wild‐type RCC2 overexpression (Figure 6C). Additionally, we analyzed the impact of RCC2K124lac on MAD2L1, compared to wild‐type RCC2, the K124R mutant did not significantly activate MAD2L1 RNA expression (Figure 6D). These data demonstrate that RCC2K124lac promotes breast cancer proliferation in vitro and in vivo.

We previously showed that RCC2 regulates MAD2L1 expression through SERBP1 interaction. However, the role of lactylation in this process was unclear. It has been reported that lactylated lysines could be situated at protein interaction interfaces, thereby affecting protein binding. For instance, lactylation of NBS1 is essential for the formation of the NBS1‐MRE11 complex.^[^ ^6^ ^]^ Since RCC2 interacts with SERBP1 via its N‐terminal domain (Figure 4I) and K124 is also located in N‐terminal, we hypothesized that RCC2 lactylation affects RCC2‐SERBP1 complex formation. Using the RCC2 and SERBP1 PDB structure, we performed docking analysis by introducing or omitting a lactyl group at K124, superimposing RCC2 structures onto SERBP1 (Figure 6E). We found that K124 did not appear to be at the interaction interface but rather adjacent to the interaction zone. Interestingly, lactyl group introduction significantly decreased complex binding energy and increased hydrogen bonds and salt bridges (Figure S4, Supporting Information). This suggests that K124 lactylation stabilizes the complex and reduces dissociation. We further verified in the cells that lactylation indeed affects the interaction between RCC2 and SERBP1. The K124R mutation reduced SERBP1 binding to RCC2 in MDA‐MB‐231 and HEK293T cells (Figure 6F,G), while NaLa treatment enhanced the interaction (Figure 6H,I). Notably, NaLa treatment indeed enhances the co‐localization of RCC2 and SERBP1 within the nucleus (Figure 6J), supporting lactylation modifications that facilitate RCC2 to recruit free SERBP1. We also assessed the influence of upstream KAT2A on the complex, and overexpression of KAT2A increased the RCC2‐SERBP1 interaction as well (Figure 6K). These data confirm that RCC2 lactylation facilitates free SERBP1 recruitment.

### 2.7 Small Molecule Drugs Inhibit RCC2K124lac and Breast Cancer Proliferation

**Figure 7:**
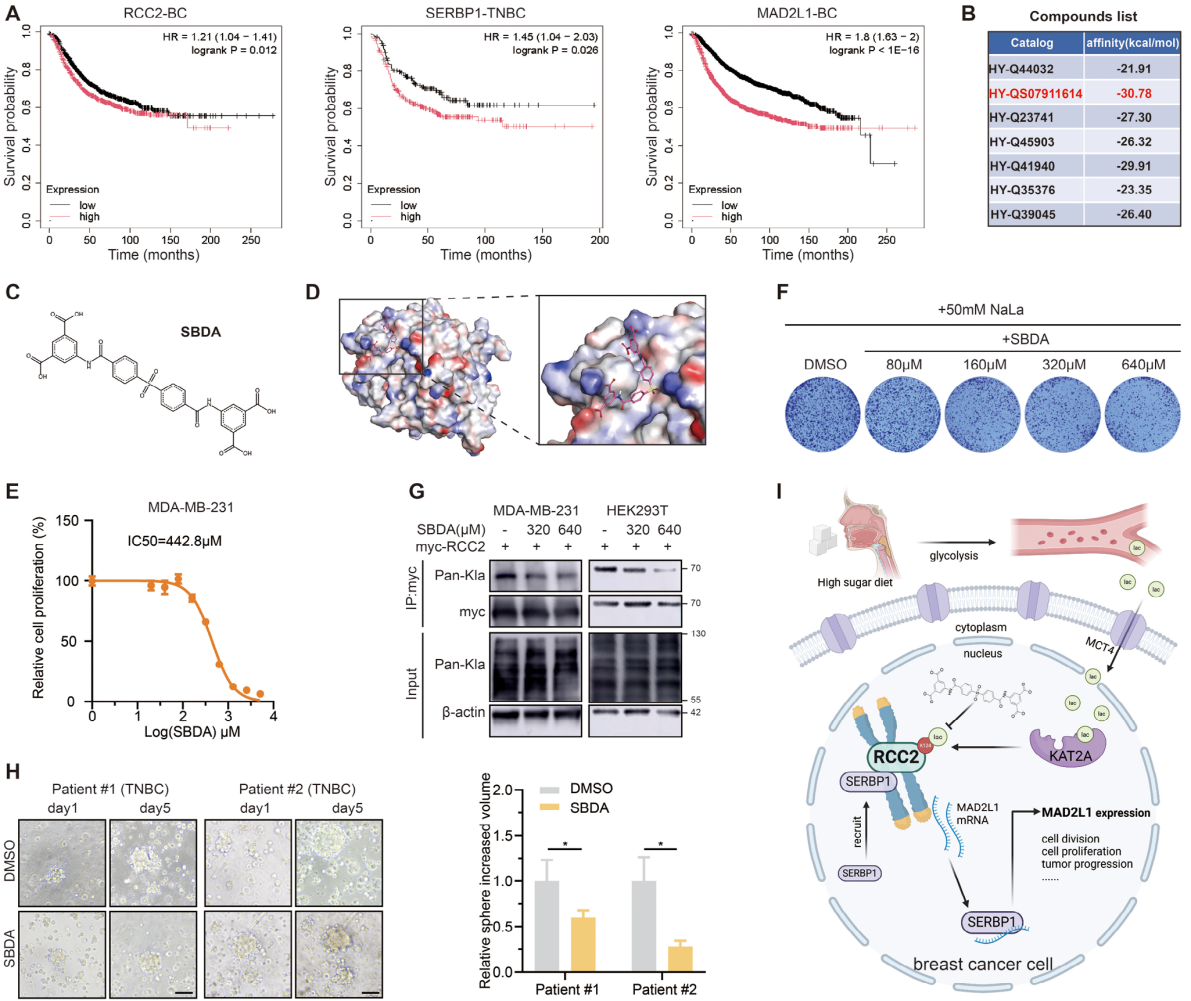
SBDA inhibits breast cancer cell RCC2 lactylation. A) The expression of RCC2, SERBP1, and MAD2L1 genes is correlated with the survival prognosis of breast cancer patients in the K‐M database (https://www.kmplot.com). B) The binding free energy of 7 candidate compounds docked to the RCC2 K124 active pocket. C) The chemical structure of SBDA. D) A docking visualization of SBDA with the active pocket of RCC2. E) MTT assays were used to assess the proliferation capacity of MDA‐MB‐231 cells after 24 h gradient concentration SBDA treatment (IC50 value was shown). F) Colony formation assays were used to assess the proliferation capacity of MDA‐MB‐231 cells after 24 h gradient concentration SBDA treatment under 50 mm NaLa. G) Exogenous RCC2 lactylation level was detected after 24 h SBDA treatment. H) The effect of 500 µm SBDA treatment on the growth of organoid cell spheres from breast cancer patients was observed by light microscopy (Shows images on day 1 and 5). Scale bars, 200 µm. I) The schematic diagram of the potential mechanism in this study. We created the images using the Biorender (https://www.biorender.com). Data are presented as mean ± SEM. *p < 0.05, **p < 0.01, ***p < 0.001, ****p < 0.0001.

Subsequently, we contemplated the clinical significance of our findings. We explored the breast cancer prognostic relevance of the genes primarily involved in this study using the K‐M database. Elevated expression levels of RCC2, SERBP1, and MAD2L1 were positively correlated with poor prognosis and shorter survival times in breast cancer patients (**Figure** **7**A), suggesting that they may serve as biomarkers for clinical prognosis assessment. Given that RCC2 lactylation activates the MAD2L1 pathway and promotes breast cancer cell proliferation, it may represent a promising therapeutic target. Subsequently, we further explored the potential druggability of the binding pocket near RCC2 K124. Specifically, we utilized a commercial compound library containing 50000 drug‐like small molecules (HY‐L901 from MCE) to screen for potential RCC2 lactylation antagonists relying on the Autodock‐Vina docking. We identified seven candidates with relatively low binding energies (Figure 7B), among which 5,5′‐((4,4′‐Sulfonylbis(benzoyl))bis(azanediyl)) diisophthalic acid (abbreviated as SBDA) had the lowest binding energy. The molecular formula of SBDA is symmetric (Figure 7C), and it interacts effectively with the amino acid residues near the K124 binding pocket (Figure 7D). Furthermore, we investigated the inhibitory effect of SBDA on cell proliferation. Due to the lack of prior research supporting our findings, we initially determined the 50% inhibitory concentration (IC50) of SBDA in MDA‐MB‐231 cells. At low concentrations (<10 µm), the inhibitory effect of SBDA on cell proliferation was nearly undetectable (data not shown). However, increasing the abundance of SBDA in the culture medium results in a concentration‐dependent inhibition of cell proliferation, with an IC50 value of 442.8 µm (Figure 7E). Under conditions of exogenous lactate addition, SBDA also inhibited cell colony formation in a concentration‐dependent manner (Figure 7F). To exclude the possibility that the antitumor effects of SBDA are attributed to its cytotoxicity, we performed the following analyses post‐treatment. Cell cycle assay revealed SBDA‐mediated G2/M phase arrest (Figure S5A, Supporting Information). Calcein/PI co‐staining showed no significant increase in dead cells following SBDA treatment (Figure S5B, Supporting Information). JC‐1 fluorescence analysis demonstrated SBDA treatment did not result in mitochondrial dysfunction (Figure S5C, Supporting Information). These findings indicate SBDA's antitumor effects stem from anti‐proliferation activity rather than cytotoxicity. SBDA treatment (320 and 640 µm) partially inhibited RCC2 lactylation levels in two cells (Figure 7G), while did not inhibit the lactylation of the RCC2 K124R mutant and the global protein lactylation (Figure S5D, Supporting Information). As anticipated, SBDA treatment significantly reduced MAD2L1 mRNA expression (Figure S5E, Supporting Information). These evidences support SBDA inhibits tumor cell proliferation via targeting RCC2 lactylation. To assess the efficacy of SBDA in a physiologically relevant tumor model, we treated tumor organoids derived from two TNBC patient samples with SBDA and observed a significant inhibition of spheroid formation (Figure 7H). These findings indicate that SBDA inhibits breast cancer cell proliferation by suppressing RCC2 lactylation, demonstrating its antitumor efficacy across different models.

## 3 Discussion

The Warburg effect, characterized by excessive lactic acid production, is common in most tumors.^[^ ^4^ ^]^ Although lactic acid's role in tumor growth is well‐documented, lactate‐mediated epigenetic regulation in breast cancer progression remains poorly understood.

MAD2L1 regulates cell division by monitoring chromosome alignment during metaphase.^[^ ^10^ ^]^ The MAD2L1 signaling pathway is aberrantly activated in various tumors. Dysregulation of MAD2L1 mediates increased tumor susceptibility,^[^ ^11^ ^]^ rapid tumor progression,^[^ ^36^ ^]^ and resistance to chemotherapy.^[^ ^37^ ^]^ MAD2L1 expression and activity are regulated by multiple factors. As a target gene of the transcription factor TEAD4, MAD2L1 is subject to transcriptional regulation and mediates the malignant phenotype of colorectal cancer.^[^ ^38^ ^]^ In cholangiocarcinoma, TBX3 inhibits progression by downregulating MAD2L1.^[^ ^38^ ^]^ Previous studies have reported that a high‐sugar diet mediates the upregulation of MAD2L1 in murine pancreatic tumors.^[^ ^12^ ^]^ Our research reveals that lactate, a product of anaerobic glycolysis, is a direct culprit in the activation of MAD2L1, although MAD2L1 protein does not serve as a direct substrate for lactylation modifications.

In this study, we reveal RCC2 has a novel role in recruiting RNA‐binding proteins and regulating RNA stability. Specifically, KAT2A mediates RCC2 lactylation at K124, which triggers recruitment of free SERBP1 via the N‐terminal domain of RCC2 and leads to the formation of the initial SERBP1‐MAD2L1 complex. SERBP1 directly binds and stabilizes MAD2L1 mRNA, ultimately upregulating MAD2L1 protein expression. Under high‐glucose culture conditions, the lactylation of RCC2 activates MAD2L1 expression and promotes the proliferation of breast cancer cells (Figure 7I), elucidating the intriguing phenomenon observed in mice.^[^ ^12^ ^]^ We hypothesize that RCC2 as a substrate protein localized on chromatin, recruits free RBPs after being lactylated, facilitating the spatial interaction between RBPs and pre‐mRNA to form a complex. Subsequent de‐lactylation of RCC2 may promote the dissociation of this complex and its nuclear exportion, thereby mediating mRNA maturation and translation.

This study has certain limitations. First, RCC2K124lac as a potential target for SBDA therapy in breast cancer, requires enhanced molecular specificity. SBDA's oral safety and the appropriate dosage for in vivo use warrant further investigation. Second, numerous RBPs were detected in the RCC2 interactome group, yet the interaction patterns between RCC2 and the remaining RBPs remain to be elucidated. Third, KAT2A as a newly discovered lactyltransferase,^[^ ^32^ ^]^ warrants further investigation into its biological functions. This includes exploring the differences in lactyl substrate specificity between KAT2A and known transferases such as KAT8 and AARS1,^[^ ^25^, ^39^ ^]^ as well as the competitive effects in mediating various acyl modifications.^[^ ^30^ ^]^

In summary, our study elucidates the mechanism through which MAD2L1 expression is enhanced in murine tumors associated with a high‐sugar diet. This suggests that lactate molecules, which are often regarded as metabolic waste in the tumor microenvironment, may influence cellular signal transduction and proliferation phenotypes through protein modifications. Furthermore, our findings proposes an innovative therapeutic approach that involves targeting RCC2 lactylation to inhibit the rapid proliferation of breast cancer cells within a high‐lactate microenvironment.

## 4 Experimental Section

### Tissue Samples

Breast tumor samples, along with the corresponding adjacent normal tissues from patients with breast cancer, were acquired by the Department of Breast and Thyroid Surgery at Shanghai Tenth People's Hospital, affiliated with Tongji University in Shanghai, China. The institutional ethics review boards at Shanghai Tenth People's Hospital approved the informed consent obtained from all study participants. The methodologies implemented in this research adhered to the ethical principles delineated in the Helsinki Declaration.

### Cell Culture, Transfection, and Drug Intervention

The HEK293T cell, normal breast epithelial cell MCF‐10A, and the breast cancer cell lines MDA‐MB‐231, BT‐549, MCF‐7, SKBR3, and BT‐474 were purchased from the Chinese Academy of Sciences (Shanghai, China) in 2022. MDA‐MB‐231, MCF‐7, BT‐474, and HEK293T cells were cultured in high glucose or low glucose Dulbecco's Modified Eagle's Medium (DMEM) (from Gibco, USA) supplemented with 10% Fetal Bovine Serum (FBS) (from Gibco, USA), 100x penicillin and streptomycin mixture solution (from Enpromise, China). BT‐549 cells were grown in the RPMI‐1640 Medium (from Gibco, USA). MCF‐10A cells were grown in the mammary epithelial basal medium (from Gibco, USA). SKBR3 cells were grown in McCoy's 5A medium (from Prisella, China). All the aforementioned cell lines were cultured at 37 °C with a 5% CO2 atmosphere. The Lipo8000 transfection reagent (from Beyotime, Shanghai, China) was utilized to introduce various knockdown and overexpression plasmids into the cell line. Initially, a combination of serum‐free DMEM medium, lipo8000, and the plasmid was prepared by the manufacturer's guidelines. Following this, the resultant mixture was applied to the culture medium of cells at about 80% confluence. After an incubation period of 8 h, the medium was replaced with a fresh complete culture medium. Flag‐MAD2L1 plasmid was kindly provided by Dr. Wang from Shanghai Tenth People's Hospital. The empty vector and other wild‐type overexpression plasmids were purchased as stock (from MiaoLingBio, Wuhan, China), the Myc‐tagged truncated RCC2 sequence plasmids and all RCC2 lysine mutant plasmids were obtained from MiaoLingBio. The Knockdown plasmids were synthesized by Generay Company (Shanghai, China) using pLKO.1‐EGFP as the vector. The target sequences for sh‐NC were 5′‐GGAGCGCGTCAAACTTGAA‐3′ and 5′‐ACACGTCCGAACATACTAC‐3′, 5′‐CAACTCAGATGGGAAGTTCAT‐3′ for sh‐RCC2 refer to the previous article.^[^ ^40^ ^]^ The target sequences for sh‐MAD2L1 were 5′‐GATTGGTTATACAAGTGTTCAT‐3′ and 5′‐CGTTCATTTACTACTACAATCT‐3′. The target sequences for sh‐KAT2A were 5′‐GCTGAACTTTGTGCAGTACAA‐3′ and 5′‐CATGTCTGTTCACAAGGAAGA‐3′ referring to the previous research.^[^ ^41^ ^]^ Cells were exposed to 50 mm NaLa (HY‐B2227B, MCE) for 24 h to increase global lactylation level, and 10 mm 2‐DG (HY‐13966, MCE) for 24 h to inhibit glycolysis. SBDA (HY‐QS07911614, MCE) was diluted to various working concentrations and the cells were treated for 24 h.

### MTT and Colony Formation

Breast cancer cells were cultured in a six‐well plate and subsequently transfected with plasmids or drugs treated for a 24‐h. Following this, the cells underwent trypsinization, and cell density was assessed to determine the requisite number of cells for further experimentation. MTT analysis involved seeding 2000 cells per well in a 96‐well plate configuration. The characterization of the cells was conducted following the manufacturer's protocols, utilizing an MTT assay kit (from Sigma, California). The optical density was recorded at 490 nm using a microplate reader at time points of 24, 48, 72, and 96 h. For the determination of drug IC50 values, 8000 cells were resuspended in a culture medium containing gradient concentrations of the drug, followed by a 24‐h incubation for optical density assessment. In the colony formation assay, 1000 cells were planted in each well of six‐well plates until cell clusters formed. Post‐rinsing the colonies twice with cold PBS, the cells were stained with 0.1% crystal violet and preserved in 75% ethanol for colony visualization. The colonies were subsequently counted and photographed.

### Animal Experiments

The sh‐NC, sh‐RCC2, sh‐MAD2L1, sh‐NC+vector, sh‐RCC2+vector, sh‐RCC2+RCC2 wt., and sh‐RCC2+RCC2 K124R stable MDA‐MB‐231 cell lines were established via puromycin selection (from Beyotime, ST551) following lentiviral transduction. Four‐week‐old female BALB/c nude mice were procured from SLAC (Shanghai, China) and assigned to various experimental groups at random (*n* = 4 per group). The cells were suspended in 150µl of DMEM devoid of fetal bovine serum and administered to the mice through subcutaneous injection, with each mouse receiving 1 × 10^6^ cells. For the in vivo experiments, sodium lactate was administered intraperitoneally to the mice at a dose of 120 mg kg^−1^ once a day, following previous publications.^[^ ^28^ ^]^ After 20 days, the mice were euthanized, and the xenograft tumors were harvested.

### Protein Separation

To isolate the total cell proteins, a phosphatase inhibitor (50X) and a protease inhibitor (PMSF 100X) were introduced to the RIPA buffer. The cells were permitted to lyse on ice after the addition of this mixture. After a 30‐min incubation, the proteins were collected, and the supernatant was obtained through 10 min 15 000 g min^−1^ centrifugation. The supernatant was then combined with the loading buffer and subjected to heating at 100 °C for 10 min. For the breast tumor samples, tissue comparable to the size of a soybean was excised using scissors, followed by the addition of lysis buffer at a temperature of 4 °C, ensuring complete homogenization of the sample. The subsequent procedures adhered to the established protocol for protein extraction from cellular sources. Based on the manufacturer's instructions, isolate and extract the proteins from the cytoplasmic and nuclear fractions (P0027, Beyotime, China).

### Immunoprecipitation

The cellular lysate was prepared using a Co‐IP buffer that contained both protease and phosphatase inhibitors. Following a 10‐min centrifugation at 12 000 g, the supernatant was collected and incubated overnight with protein A/G magnetic beads (from Absin, abs955, China) in the presence of specific antibodies at 4 °C. On the subsequent day, the beads were subjected to five wash cycles utilizing the IP buffer. Elution of the immunoprecipitates was accomplished by heating the samples in a 1xSDS loading buffer.

### Western Blot

SDS‐PAGE was employed to fractionate protein samples, which were subsequently transferred onto a nitrocellulose membrane utilizing Rapid Transfer Buffer (from New Cell & Molecular Biotech, WB4600, China). Following a blocking step with 5% BSA, the membrane was incubated overnight with the primary antibody, and then exposed to the secondary antibody for 1 h at room temperature. The membrane was thoroughly washed with TBST for 10 min in three separate rounds. Ultimately, the blots were visualized through chemiluminescence. A detailed list of the antibodies used can be found in Table S1 (Supporting Information).

### In Vitro Lactylation Assay

This experiment references previous literature.^[^ ^28^ ^]^ In reaction buffer with 20 mm lactyl‐CoA, the HA‐tagged KAT2A proteins that were isolated from HEK293T cells were treated with the GST fusion RCC2 proteins. The reactions were incubated for 30 min at 30 °C. Following the addition of SDS loading buffer, the reaction was boiled for 5 min at 100 °C. The obtained samples were subsequently western blot analyzed.

### Silver Staining and Mass Spectrum

The MDA‐MB‐231 cell line was transfected with myc‐RCC2 plasmids for a duration of 48 h, followed by treatment with MG132 at 25 mm for 3 h. Subsequently, immunoprecipitation of the cell lysates was conducted using antibodies targeting the myc epitope tag. For silver staining, the Protein Silver Stain Kit (P0017S, Beyotime) was employed. Mass spectrometry (carried out by oebiotech company, Shanghai, China) was utilized to identify proteins that interact with RCC2 independently. The detailed data is listed in Table S3 (Supporting Information).

### Lactate Level, Cell Cycle, Calcein/PI, and JC‐1 Assay

Detect the intracellular lactate levels according to the manufacturer's instructions (KTB1100, Abbkine). Cell cycle assay was performed according to the manufacturer's instructions (40301ES50, Yeasen). Calcein/PI assay was performed according to the manufacturer's instructions (C2015S, Beyotime). JC‐1 assay was performed according to the manufacturer's instructions (M8650, Solarbio, China).

### Quantitative Real‐Time PCR

Following the manufacturer's instructions, total RNA was isolated from cells using the TRIzol reagent (from Beyotime, Shanghai, China). Single‐stranded cDNA was produced with the use of a cDNA Synthesis Kit (from Abclonal, China). qRT‐PCR was carried out using the SYBR Green Master Mix (from Abclonal, Shanghai, China, RK21203). The primers used are listed in Table S1 (Supporting Information).

### RNA Immunoprecipitation and Agarose Gel Electrophoresis Assay

RIP assays were performed using the BersinBioTM RNA immunoprecipitation (RIP) Kit (Bes5101, BersinBio, China) according to the manufacturer's instructions. Before immunoprecipitation, Flag‐SERBP1 plasmids were transferred into the IgG and Ip groups. To conduct RIP tests, anti‐Flag antibodies (from ABclonal, Shanghai, China, AE005) and appropriate control IgG antibodies from BersinBio were used. The resulting RNA was reversely transcribed and amplified by PCR with specific primers. The obtained products were subjected to 1.5% agarose gel electrophoresis and strip images were captured by the Gel Doc XR + imager (Bio‐Rad, Hercules, CA, USA).

### Actinomycin D Assay

Actinomycin D (from Merck, Germany) was administered to MDA‐MB‐231 and BT‐549 cell lines at a concentration of 2 mg mL^−1^ to inhibit transcription. Subsequently, the isolated RNAs from the treated cells were subjected to analysis via qRT‐PCR.

### Immunofluorescence Staining

Cells were cultivated on glass coverslips within 24‐well culture plates. Following a wash with PBS, the cells were fixed for 10 min, subsequently permeabilized using 0.1% Triton X‐100, and blocked with 5% BSA for 30 min. The cells were then subjected to staining with specific antibodies. MDA‐MB‐231 and BT‐549 cells, which were transfected with respective plasmids, received staining with the anti‐Flag (Mouse) and anti‐Myc (Rabbit) antibodies. Secondary antibodies conjugated with DyLight 488 (AS037, Abclonal, China) and DyLight 594 (AS039, Abclonal, China) were applied. Nuclei were stained with DAPI (G1407‐25ML, Servicebio, China). After capturing images, the resulting photographs were analyzed using ImageJ.

### Immunohistochemistry (IHC)

Patient tissue specimens were stained with anti‐Pan‐Kla (PTM‐1401, PTMbio, China) after fixation, dehydration, embedding, and sectioning.Ki‐67 staining of the animal tissues was performed by Servicebio. The images were captured and subsequently analyzed using ImageJ software. For each immunohistochemistry (IHC) slide, five distinct fields at a magnification of 200X were examined to determine the percentage of positively stained cells. The IHC score was then calculated as the product of the positive cell percentage and the intensity of the positive staining observed.

### Patient‐Derived Organoid

Tumor tissues from breast cancer patients were collected by the Department of Breast and Thyroid Surgery. Fresh specimens of tumor tissue were stored on ice during transportation to the laboratory. Erythrocytes were washed, digested, and removed by the reagent manufacturer's specifications (abs9446, Absin, China). The resulting cell suspension was combined with matrix gel and subsequently seeded into 24‐well plates. A specialized culture medium was introduced, and incubation occurred at 37 °C. The dimensions of cell clusters in both the drug treatment and control groups were examined using a light microscope.

### Molecular Docking and Virtual Screening

The protein structure was retrieved from UniProt and the full‐length AlphaFold predictive structure of SERBP1 (UniProt ID: Q8NC51) was selected as the ligand‐protein, while the full‐length AlphaFold predicted structure of RCC2 (UniProt ID: Q9P258) was selected as the receptor protein. AutoDockTools was used for dock analysis, during which all protein files were converted into PDBQT format, with all water molecules removed and polar hydrogen atoms added. The grid box was positioned at the center to facilitate molecular movement and encompass the protein's domain. The binding energy of the protein complex when RCC2 with/without lactylation was computed using the MM/GBSA method on the HawkDock server. For virtual screening, centering on site 124 lysine at RCC2 protein, the binding region was enlarged to the maximum to determine its active pocket. Docking analysis with RCC2 active pockets was performed using a commercialized library of drug compounds containing more than 50000 small molecules (HY‐L901, MCE). The virtual screening summary is listed in Table S2 (Supporting Information).

### Statistical Analysis

Data obtained from three independent experimental trials were presented as mean ± standard error of the mean (SEM). Statistical evaluations were conducted using GraphPad Prism 8 (GraphPad, San Diego, CA) and Microsoft Excel. A two‐tailed Student's *t*‐test was employed to assess statistical significance between two groups, while a one‐way ANOVA was used for comparisons involving more than two groups. The correlations between Pan‐Kla levels and the clinicopathologic features of patients were determined using the Chi‐square test or Fisher's exact test. The threshold for statistical significance across all analyses was established at *p* < 0.05.

## Conflict of Interest

The authors declare no conflict of interest.

## Author Contributions

Z.B.W., P.Y.H., and Q.F.Y. contributed equally to this study. Z.B.W. and F.L. designed the study. Z.B.W. and P.Y.H. conducted the experiments and wrote the manuscript. Q.F.Y., L.D.Y., and Y.D.R analyzed the data. Z.W.F. and W.X.H. directed the details of the experiment and supervised the study. Y.B.L., Z.S., Z.B.A., and W.Y.Y. collected the breast cancer tissues. F.L. and Y.D.R. provided funding support. All authors have reviewed and approved the paper.

## Ethics Approval and Consent to Participate

This study was approved by the Ethics Committee of the Shanghai Tenth People's Hospital (No.2020‐KN174‐01). All animal experiments were approved by the Animal Care and Use Committee of the Shanghai Tenth People's Hospital (No. SHDSYY‐2023‐0600).

## Consent for Publication

All co‐authors have given their consent to the submitted version of the manuscript for publication.

## Supporting information



Supporting Information

Supplemental Table 2

Supplemental Table 3

Supplemental Table 3

## Data Availability

The data that support the findings of this study are available from the corresponding author upon reasonable request.
