# Supplementary material for: High Sugar Induced RCC2 Lactylation Drives Breast Cancer Tumorigenicity Through Upregulating MAD2L1
Source: Adv Sci (Weinh). 2025 Mar 27;12(21):2415530. doi: 10.1002/advs.202415530 (PMC12140329; doi:10.1002/advs.202415530)
Supplement: Supplementary file 1 — Supporting Information [file ADVS-12-2415530-s003.pdf]

ADVANCED SCIENCE
Open Access

Supporting Information

for *Adv. Sci.*, DOI 10.1002/advs.202415530

High Sugar Induced RCC2 Lactylation Drives Breast Cancer Tumorigenicity Through Upregulating MAD2L1

*Bowen Zheng, Yunhao Pan, Fengyuan Qian, Diya Liu, Danrong Ye, Bolin Yu, Seng Zhong, Wenfang Zheng, Xuehui Wang, Baian Zhou, Yuying Wang and Lin Fang**

Supplementary Figure S1-S5

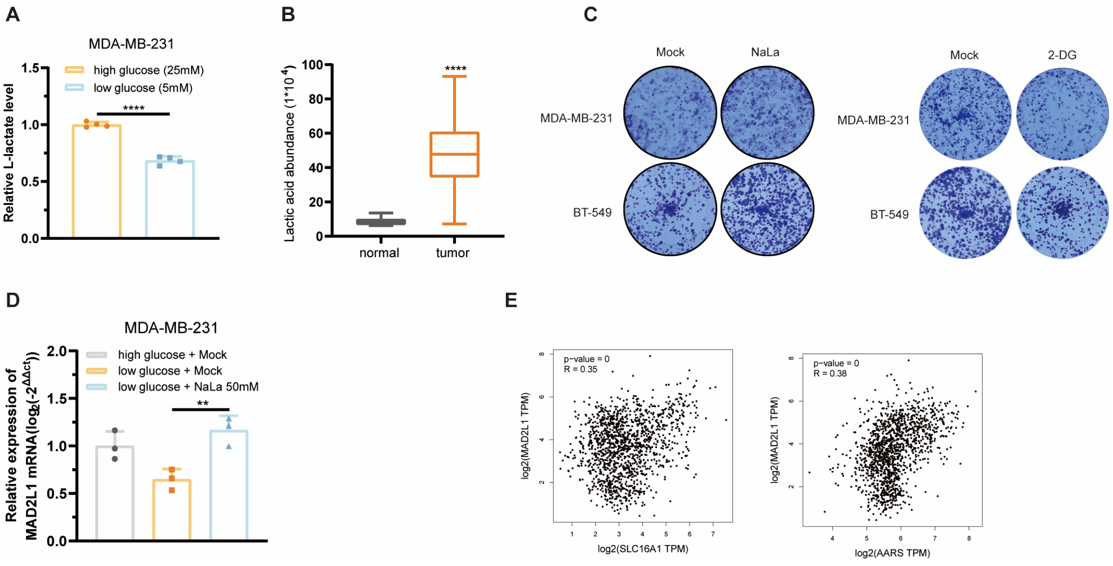

Fig.S1 A Detection of intracellular lactate level under high-glucose (25mM) or low-glucose (5mM) culture conditions in MDA-MB-231. **B** The lactate levels in breast cancer tissues and adjacent normal tissues within our own cohort. **C** Colony formation assays were used to assess the proliferation capacity of the cell with or without 2-DG and NaLa treatment. **D** PCR assays of MAD2L1 expression level in MDA-MB-231 cell with the given conditions. **E** Correlation analysis of the expression of MAD2L1, SLC16A1 and AARS in breast cancer tissues from the TCGA database. (http://gepia.cancer-pku.cn/). Data are presented as mean ± SEM. *p < 0.05, **p < 0.01, ***p < 0.001, ****p < 0.0001.

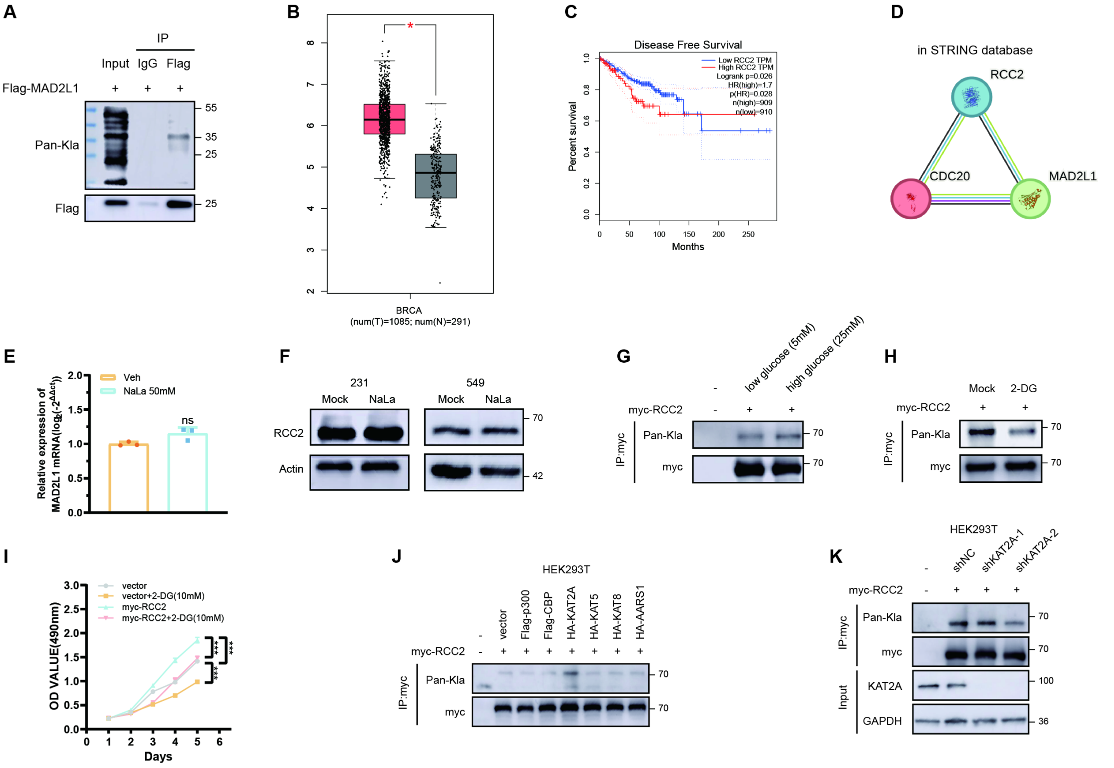

Fig.S2 A Detection of exogenous MAD2L1 protein lactylation (near 25kD) in MDA-MB-231 cell. **B** RCC2 mRNA expression in breast cancer and normal tissue based on UALCAN database (https://ualcan.path.uab.edu/). **C** RCC2 expression correlated with breast cancer patients' disease free survival in TCGA database. **D** The analysis from STRING database suggests that RCC2 correlated with MAD2L1. **E** PCR assays of RCC2 expression level in MDA-MB-231 with or without 50mM NaLa treatment. **F** Western blot analysis of RCC2 expression level with or without 50mM NaLa treatment. **G** Lactylation level of exogenous RCC2 under high-glucose (25mM) or low-glucose (5mM) culture conditions in MDA-MB-231. **H** Lactylation level of exogenous RCC2 with or without 10mM 2-DG treatment in MDA-MB-231. **I** MTT assays was used to assess the proliferation capacity of the MDA-MB-231 cell when RCC2 was overexpression with or without 10mM 2-DG treatment. **J** Exogenous RCC2 lactylation level when a series of acyltransferases were overexpressed in HEK293T. **K** Exogenous RCC2 lactylation level when KAT2A was knocked down in HEK293T. Data are presented as mean ± SEM. *p < 0.05, **p < 0.01, ***p < 0.001, ****p < 0.0001.

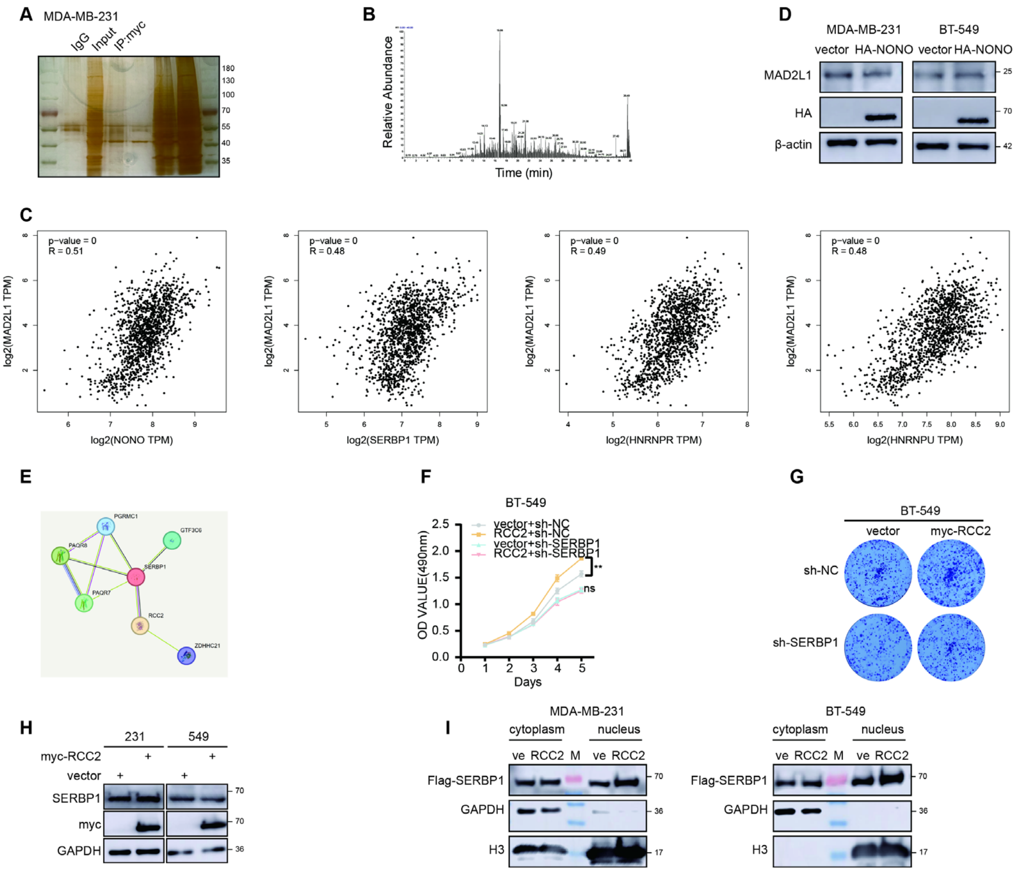

Fig.S3 A and B Silver staining and Mass Spectrometry assays of proteins pulled down by IP experiments in MDA-MB-231 cell. **C** Correlation analysis of the expression of RBPs and MAD2L1 in breast cancer tissues from the TCGA database (http://gepia.cancer-pku.cn/). **D** Western blot analysis of MAD2L1 expression level when NONO was overexpression. **E** The analysis from STRING database suggests that RCC2 may interact with SERBP1. **F and G** MTT and colony formation assays were used to assess the proliferation capacity of the SERBP1-silenced BT-549 cell with or without RCC2 overexpression. **H** Western blot analysis of SERBP1 expression level when RCC2 was overexpression. **I** Western blot analysis of the expression level of exogenous SERBP1 in nucleus and cytoplasm when RCC2 was overexpression. Data are presented as mean ± SEM. *p < 0.05, **p < 0.01, ***p < 0.001, ****p < 0.0001.

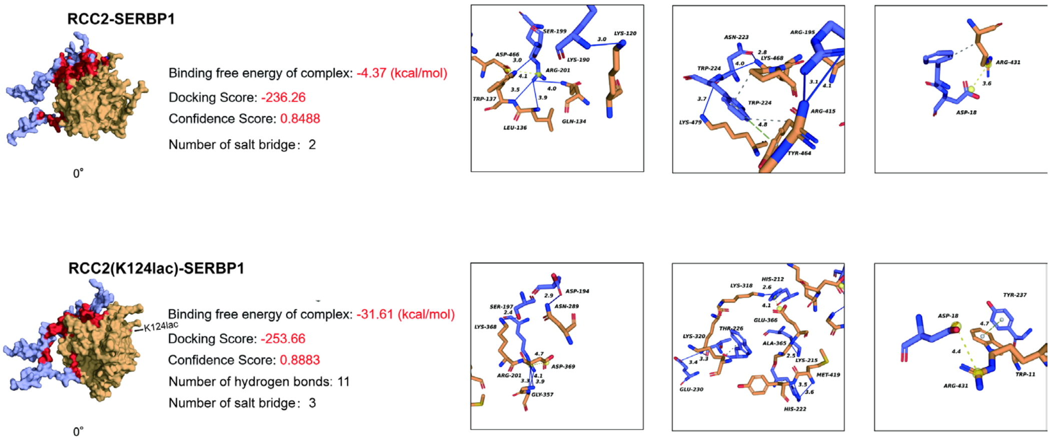

Fig.S4 The docking of RCC2 protein (UniProt ID: Q9P258) and SERBP1 protein (UniProt ID: Q8NC51) is when the site 124 lysine in RCC2 is either modified or unmodified by a lactyl group. This includes the free energy, the number of hydrogen bonds and salt bridges of the two complexes, as well as the bonding details observed from different angles.

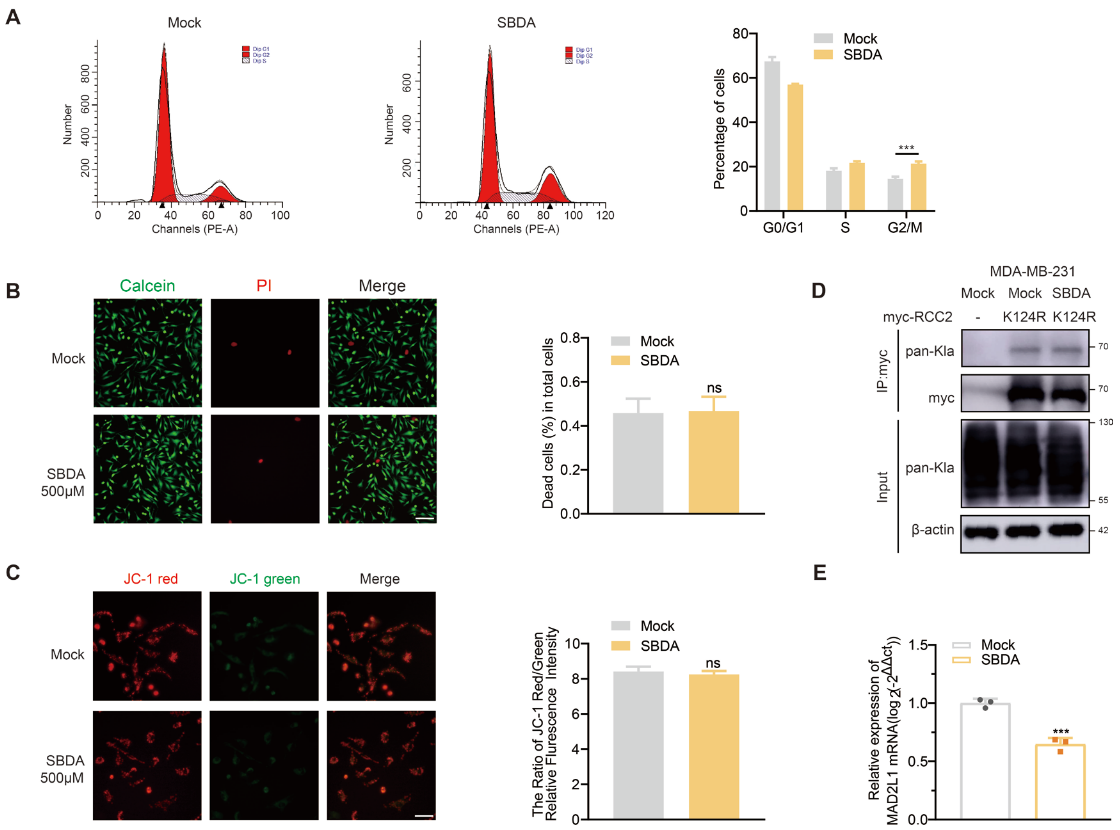

Fig.S5 A Cell cycle assay in MDA-MB-231 cells treated with 500μM SBDA. **B** Calcein/PI assay in MDA-MB-231 cells treated with 500μM SBDA. Green indicates live cells, while red signifies dead cells. Scale bars, 80 μm. **C** JC-1 staining assay in MDA-MB-231 cells treated with 500μM SBDA. Scale bars, 20 μm**. D** Exogenous RCC2 lactylation level when transfected RCC2 K124R plasmids with or without 500μM SBDA treatment. **E** PCR assays of MAD2L1 expression level in MDA-MB-231 cells treated with 500μM SBDA. Data are presented as mean ± SEM. *p < 0.05, **p < 0.01, ***p < 0.001, ****p < 0.0001.
