# Supplementary material for: High Sugar Induced RCC2 Lactylation Drives Breast Cancer Tumorigenicity Through Upregulating MAD2L1
Source: Adv Sci (Weinh). 2025 Mar 27;12(21):2415530. doi: 10.1002/advs.202415530 (PMC12140329; doi:10.1002/advs.202415530)
Supplement: Supplementary file 4 — Supplemental Table 3 [file ADVS-12-2415530-s001.pdf]

ADVANCED SCIENCE
Open Access

Supporting Information

for *Adv. Sci.*, DOI 10.1002/advs.202415530

High Sugar Induced RCC2 Lactylation Drives Breast Cancer Tumorigenicity Through Upregulating MAD2L1

*Bowen Zheng, Yunhao Pan, Fengyuan Qian, Diya Liu, Danrong Ye, Bolin Yu, Seng Zhong, Wenfang Zheng, Xuehui Wang, Baian Zhou, Yuying Wang and Lin Fang**

Supplemental Tables 1

Primers used in this research

Gene	Sequences(5’-3’)
h-MAD2L1-F	TTGAGTGTGACAAGACTGCAAAAG
h-MAD2L1-R	CAGTGGCAGAAATGTCACCGTAG
h-GAPDH-F	GTCTCCTCTGACTTCAACAGCG
h-GAPDH-R	ACCACCCTGTTGCTGTAGCCAA
h-RCC2-F	CCAAACGTGGTTGTACGAGACG
h-RCC2-R	GGACCATCTCATCCTTCTGCTC
h-SERBP1-F	AGAAAGGCGACCACCTCGTGAA
h-SERBP1-R	ACCTCTTCCAAGACCACCACGA
h-beta-actin-F	CACCATTGGCAATGAGCGGTTC
h-beta-actin-R	AGGTCTTTGCGGATGTCCACGT

Antibodies used in this research

Protein	cat	company	Concentrations for western blot	Concentrations for immunofluorescence
Anti-RCC2 (Mouse)	sc-514340	Santa cruz	1:400	
Anti-RCC2 (Rabbit)	A13105	abclonal	1:1000	
Anti-RCC2 (Rabbit)	ER64774	HUABIO	1:1000	
Anti-SERBP1	10729-1-AP	proteintech	1:1000	
Anti-MAD2L1	10337-1-AP	proteintech	1:1000	
Anti-MAD2L1	ET7106-69	HUABIO	1:1000	
Anti-L-Lactyllysine	PTM-1401RM	PTMBio	1:1000	
Anti-Myc-tag (Mouse)	60003-2-Ig	proteintech	1:10000	
Anti-Myc-tag (Rabbit)	16286-1-AP	proteintech	1:10000	1:200
Anti-Flag-tag (Mouse)	66008-4-Ig	proteintech	1:10000	1:1000
Anti-Flag-tag (Rabbit)	20543-1-AP	proteintech	1:10000	
Anti-HA-tag (Mouse)	AE008	abclonal	1:5000	
Anti-HA-tag (Rabbit)	AE105	abclonal	1:5000	
Anti-Control IgG (Mouse)	AC011	abclonal		
Anti-Control IgG (Rabbit)	AC005	abclonal		
Anti-GAPDH	HRP-60004	proteintech	1:50000	
Anti-Histone H3	A2348	abclonal	1:10000	
Anti-beta-actin	66009-1-Ig	proteintech	1:50000	1:1000
HRP Goat Anti-Rabbit IgG	AS014	abclonal	1:4000	
HRP Goat	AS003	abclonal	1:4000	

Anti-Mouse IgG			
Anti-KAT8	ET7108-23	HUABIO	1:1000
Anti-KAT2A	ER63516	HUABIO	1:1000
Anti-KAT2A	66575-1-Ig	proteintech	1:2000
